# Loss of α6β4 Integrin-Mediated Hemidesmosomes Promotes Prostate Epithelial Cell Migration by Stimulating Focal Adhesion Dynamics

**DOI:** 10.3389/fcell.2022.886569

**Published:** 2022-07-07

**Authors:** Anette Schmidt, Mika Kaakinen, Tomasz Wenta, Aki Manninen

**Affiliations:** ^1^ Disease Networks Research Unit, Faculty of Biochemistry and Molecular Medicine, Biocenter Oulu, University of Oulu, Oulu, Finland; ^2^ Oulu Center for Cell-Matrix Research, Faculty of Biochemistry and Molecular Medicine, Biocenter Oulu, University of Oulu, Oulu, Finland; ^3^ Department of General and Medical Biochemistry, Faculty of Biology, University of Gdansk, Gdansk, Poland

**Keywords:** α6β4-integrins, hemidesmosome, focal adhesion regulation, prostate cancer, CRISPR/Cas9 knock-in

## Abstract

Epithelial cell adhesion is mediated by actin cytoskeleton-linked focal adhesions (FAs) and intermediate filament-associated hemidesmosomes (HDs). HDs are formed by α6β4-integrins and mediate stable anchoring to the extracellular matrix (ECM) while FAs containing β1-integrins regulate cell migration. Loss of HDs has been reported in various cancers such as prostate cancer where it correlates with increased invasive migration. Here we have studied cell migration properties and FA dynamics in genetically engineered prostate epithelial cell lines with intact or disrupted HDs. Disruption of HDs by depleting α6- or β4-integrin expression promoted collective cell migration and modulated migratory activity. Dynamic analysis of fluorescent protein-tagged FA marker proteins revealed faster FA assembly and disassembly kinetics in HD-depleted cells. FRAP analysis showed that loss of HDs correlated with faster diffusion rates of focal adhesion kinase (FAK) and vinculin in and out of FAs. These data suggest that loss of α6β4-mediated HDs promote cell migration and FA assembly dynamics by influencing the molecular diffusion rates of FAK.

## Introduction

Epithelial cells form two distinct types of integrin-mediated extracellular matrix (ECM) adhesions, focal adhesions (FAs) and hemidesmosomes (HDs). FAs are linked to the actin cytoskeleton whereas HDs are connected to the intermediate filament network (Tsuruta et al., 2011). At least two distinct types of HDs have been found. The stratified epithelium contains highly organized type I HDs composed of α6β4-integrins, plectin, tetraspanin CD151, bullous pemphigoid antigen 1 (BPAG1) and type XVII collagen (Walko et al., 2015). Simple epithelia, for example in the intestine and prostate, form less well-defined type II HDs that reportedly lack BPAG1 and type XVII collagen. Current evidence suggests that FA dynamics regulate mechanotransduction and cell locomotion (De Pascalis and Etienne-Manneville, 2017) while HDs function as stable anchoring adhesions and a prerequisite for apicobasal polarization (Walko et al., 2015; te Molder et al., 2021). The assembly of FAs and HDs is tightly coordinated in normal epithelial cells such that they form adjacent, yet non-overlapping, adhesion complexes (Myllymäki et al., 2019). While it is evident that dynamic signaling crosstalk coordinates HDs and FAs assembly the molecular mechanisms of this crosstalk are just beginning to be unraveled (Zuidema et al., 2020).

Disassembly of type I HDs in keratinocytes has been shown to promote cell migration by modulating FA dynamics (Ozawa et al., 2010). Whether type II HDs similarly inhibit FA dynamics remains incompletely understood. FAs and HDs co-exist in most normal epithelial cell types but α6β4-integrin-mediated HDs are frequently disassembled or lost in prostate cancer cells during disease progression (Allen et al., 1998; Davis et al., 2001). This is accompanied by disrupted epithelial polarity and it correlates with increased invasive capacity of prostate cancer cells (Wenta et al., 2021). Whether the loss of HDs is directly linked to increased invasiveness of prostate cancer cells remains unclear but HDs have been shown to affect FAs assembly (Wang et al., 2020) and a recently published preprint reported increased migratory capacity of α6- or β4-integrin-depleted prostate cancer cells (Wenta et al., 2021). Here we studied the effects of HDs disruption on cell migration and FAs formation and turnover in prostate epithelial cells. We found that disruption of HDs by depleting the expression of α6- or β4-integrins led to increased cell migration in the scratch wound assay. HD-depleted cells had more numerous FAs and analysis of fluorescent protein-tagged FA marker proteins revealed increased FA dynamics. HD-deficient cells efficiently formed numerous FAs that had a shorter lifetime. Loss of HDs was accompanied by faster diffusion rates of FAK, a critical FA-signaling protein, within FAs. Molecular dynamics of vinculin were also stimulated suggesting impaired FA maturation. Interestingly, the observed effects were particularly evident in β4-depleted cells suggesting a selective advantage for invasive prostate cancer cells to lose β4-subunit expression as is observed in most aggressive prostate cancer cells.

## Materials and Methods

### Cell Culture

RWPE1 (CRL-11609) and PC3 (CRL-1435) cell lines were purchased from ATCC. RWPE1 cells were cultured in Keratinocyte SFM medium (#17005042; Gibco, Thermo Fischer Scientific) supplemented with bovine pituitary extract (#13028-014) and human recombinant EGF (#PHG0311), and standard antibiotics: penicillin (100 units/ml) and streptomycin (100 μg/ml), according to the manufacturer’s protocol. PC3 cells were maintained in Ham’s F12K medium (#21127030; Gibco, Thermo Fischer Scientific) containing 10% fetal bovine serum (Gibco, Thermo Fischer Scientific) and standard antibiotics. The cells were maintained at 37°C in a humidified incubator with 5% CO_2_. All cell lines were routinely tested for *mycoplasma sp.* during the experiments.

### Plasmid Construction

The generation of α6- (gRNA: TTTTC TTTGG ACTCA GGGAA AGG targeting exon 6 of *ITGA6*) and β4-integrin (gRNA: CAACT CCATG TCCGA TGATC targeting exon 5 of *ITGB4*) -depleted RWPE1 and PC3 cells is described in (Wenta et al., 2021). For knock-ins, the HDR-based CRISPR-Cas9 approach was adopted (Nguyen et al., 2020). The schematic illustration of the protocol is presented in Figure 4A. Genomic DNA from RWPE1 was extracted using QuickExtract DNA Extraction Solution (Lucigen QE09050) and used as a template for PCR reaction to amplify the left and right homology arm flanking the start codon of *VCL* sequence. To generate mScarlet-donor plasmid (pUC19-derived) containing the left homology arm of *VCL* fused with *mScarlet* and right homology arm of *VCL* sequences the In-Fusion HD Cloning Kit (Takara Bio, 638910) was applied. The gRNA sequence for *mScarlet-VCL* knock-in (CGTAT GAAAC ACTGG CATCG) was incorporated into plentiCRISPRv2_neo vector as described above. The details of the plasmids used for vinculin, FAK and ILK overexpression in RWPE1 and PC3 are shown in Supplementary Table S1 and Hara et al., 2008; Pietilä et al., 2012; Zhang et al., 2002. All the plasmids were verified by sequencing.

### Viral Transduction

Lentiviral particles were produced as described in Wenta et al. (2021). In short, 5 μg of specific plentiCRISPRv2-gRNA, 3.75 μg psPax2 and 1.25 μg of pVSV-G plasmids were co-transfected into human embryonic kidney 293T cells (ATCC, LGC Standards GmbH, Wesel, Germany; CRL-11268) using Lipofectamine 2000 (Invitrogen, Thermo Fisher Scientific). Virus-containing supernatant was filtered using a 0.45 µM spin filter (Sartorius Minisart®). Hexadimethrine bromide (Sigma Alrich; H9268) was added to virus suspension to a final concentration of 4 µg/ml. Target cells grown were washed twice with PBS followed by the addition of 1 ml of virus suspension per 3.5 cm Ø tissue culture dish. After 2 h of incubation (5% CO_2_; 37°C), 0.5 ml of fresh culture medium was added and incubation was continued for 48 h followed by the addition of 1 μg/ml (PC3) or 0.5 μg/ml (RWPE1) of puromycin for at least 7 days to eliminate non-transduced cells. Depletion of target gene expression was confirmed by western blotting.

### Knock-In Cell Line Generation

1·10^6^ cells were electroporated with 4 µg mScarlet donor plasmid DNA using Amaxa Nucleofector™ 2b Device (Lonza) and immediately seeded in 5 ml of the complete medium on one well of a 6-well plate and incubated at 37°C, 5% CO_2_ for 6 h. Dead and non-adherent cells were discarded by washing twice with 1 ml of complete medium and attached cells were transduced using *VCL*-targeting gRNA expressing lentiviral vectors, as described above. The next day, supernatant was removed, and cells were gently washed with fresh complete medium. Antibiotic selection with G418 (Thermo Fisher Scientific) was started 48 h post-transduction and continued for at least 7 days. Finally, mScarlet-positive cells were sorted using FACS. At least 300 mScarlet-positive cells were collected for the generation of each variant. The mScarlet-*VCL* recombination was confirmed by western blotting, fluorescence microscopy and sequencing.

### Western Blotting

Nearly confluent cells were washed with PBS (Gibco) and scraped into RIPA buffer: 10 mM Tris-HCl pH 8.0, 150 mM NaCl, 0.5% SDS, 1% IGEPAL, 1% sodium deoxycholate containing 2 mM PMSF (phenylmethylsulfonyl fluoride), 10 μg/ml aprotinin, and 10 μg/ml leupeptin. Protein concentration was estimated using BCA Protein Assay Kit (Pierce) and 30 µg of protein lysate was resolved in SDS-PAGE and transferred onto a Protran pure 0.2micron nitrocellulose (Perkin Elmer). Non-specific binding was blocked by incubating membranes for 1 h in 5% skimmed milk followed by probing with specific primary antibodies (Supplementary Table S2) overnight at 4°C. Secondary antibodies conjugated with HRP and Lumi-Light Western Blotting Substrate (Roche) were used to visualize specific protein bands that were detected using Fujifilm LAS-3000 bioimaging and scientific research imaging equipment (Fuji Photo Film Co., Ltd.).

### Proliferation Assay

4·10^3^ of the indicated RWPE1 or 2·10^3^ PC3 cell variants were seeded onto the 96-wells plate in a complete culture medium. Cell proliferation was assessed using an XTT-based Cell Proliferation Kit II (Roche 11465015001). The data were collected at the indicated time points by measuring the absorbance at 450/750 nm according to the manufacturer’s instructions. All data were obtained in triplicate from two independent experiments.

### Scratch Wound Assay

8·10^4^ RWPE1 or 5·10^4^ PC3 cells were seeded onto wells of Incucyte ImageLock 96-well Plate (Essen BioScience Inc. #4379) and cultured for 24–48 h to reach full confluency. A wound was made using the Woundmaker 96 tool (Essen Bioscience Inc.) and the migration of the cells into the scratched wound area was analyzed using IncuCyte S3 Live-Cell Analysis System (Essen Bioscience Inc.).

### Cell Tracking

2.5·10^5^ RWPE1 or 1.5·10^5^ PC3 cells were seeded onto 6-well plates coated with 4.1 µg/cm^2^ fibronectin (Advanced Biomatrix) and allowed to settle for 15 h. For time-lapse imaging, cell culture plate was placed into temperature-controlled Okolab stage-top incubator (37^o^C; 5% CO_2_). Imaging was done with Olympus IX81 inverted microscope with CPlanFLN PhC 10x/0.30 objective by using the phase-contrast technique. Images were captured every 2 min for 3 h with Olympus XM10 CCD camera and Olympus CellSens software. Cell centroids were tracked in consecutive image frames by using the manual tracking plugin of Fiji processing package of Image J2 software (Schindelin et al., 2012). Cell mobility and migration characteristics were analyzed with IBIDI Chemotaxis and Migration Tool plugin (Zengel et al., 2011).

### Immunofluorescence Microscopy

For immunofluorescence cells on 35 mm glass-bottom μ-Dish (IBIDI) were washed twice with PBS and fixed with 4% PFA (paraformaldehyde) in PBS for 15 min. PFA was quenched with 100 mM glycine in PBS for 20 min followed by cell permeabilization using 0.1% Triton X100 in PBS for 15 min. Unspecific binding was blocked with 1 h incubation in 0.2% gelatin and 0.5% BSA in PBS. Samples were incubated overnight at 4°C with primary antibodies diluted in blocking buffer (Supplementary Table S2). Samples were washed four times before incubation with secondary antibodies conjugated with a fluorophore for 1 h at room temperature. Samples were washed and analyzed using Zeiss LSM 780 confocal microscope or Zeiss Cell Observer.Z1 microscope equipped with a total internal reflection fluorescence (TIRF) module. ZEN Blue software was applied to data analysis.

### Focal Adhesions Marker Analysis

The total area and the average size of FA foci were measured using Fiji ImageJ software. The same settings were applied for the analysis of all the images with a given FA-marker. Data are present as a mean ± SD of at least 30 pictures taken from random places on the IBIDI plates.

### Focal Adhesions Dynamic Analysis

2.5·10^5^ RWPE1 or 1.5·10^5^ PC3 cells were seeded on 35 mm glass-bottom μ-Dish (IBIDI) coated with 4.1 µg/cm^2^ fibronectin (Advanced Biomatrix) and analyzed after 24 h using Zeiss Cell Observer.Z1 Spinning Disc confocal microscope, ×63x oil. FAs were monitored for 3 h using the TimeLapse function with 1 min intervals. The lifetime of FAs was measured by following assembly and disassembly of single FA foci in Fiji ImageJ software (Schindelin et al., 2012). At least six random cells from every variant with ten FA foci in each were analyzed. One-way ANOVA (GraphPad Prism 8) was used to determine statistical significance. Color-coded timelapse images were generated using the Temporal-Color Code Fiji-plugin developed by Kota Miura for background-corrected time series covering a 30-min time span.

### Fluorescence Recovery After Photobleaching

2.5·10^5^ RWPE1 or 1.5·10^5^ PC3 cells with GFP/mScarlet-tagged constructs were seeded onto 35 mm glass-bottom μ-Dish (IBIDI) to reach 30% confluence and imaged after 24 h. FRAP experiment was performed using Zeiss LSM780 AxioObserver equipped with incubation chamber (37°C, 5% CO_2_), ×63x/1.40 oil DIC M27, 488-nm laser or 561-nm laser, with ZEN black software. Fluorescence was measured at low laser power 1–2% in 1s intervals. Imaging was taken at zoom factor 3 (512 × 512) with 20 prebleach scans followed by bleaching size 0.5 × 0.5 µM square ROI areas with 100% of laser power for 2-3 fluorescence tagged FA foci at the same time. The recovery of fluorescence was monitored for 4–5 min (250-330 postbleach scans) until the fluorescence recovery reached a plateau. The data were collected from at least 50 individual FAs from at least 10 cells. Additional unbleached ROIs were measured with ZEN Black to subtract background intensities and to correct fluorescence loss caused by photobleaching. Background subtraction and normalization of the intensities to the average of prebleach were calculated as in (Long et al., 2021). Mobile fractions were calculated according to M_f_ = (F_∞_—F_0_)/(F_pre_—F_0_), where F_pre_ is the average intensity before bleaching, F_0_ is the first postbleach intensity in a bleached area, F_∞_ is the postbleach intensity at a plateau. Curve fitting of the bleached size 0.5 × 0.5 µm square ROI areas mobile fractions and determination of half-life times (*t*_*1/2*_) were performed in GraphPad Prism 8 software using linear regression/one phase association algorithm.

### Statistical Analysis

Data are expressed as means ± SD of at least three independent experiments. For data following a normal distribution, One-way ANOVA followed by Sidak’s multiple comparisons test or Two-way ANOVA followed by Dunnett’s multiple comparisons test were applied. In other cases, comparative data were analyzed with Kruskal-Wallis test followed by Dunn’s multiple comparisons test as indicated using GraphPad Prism 8 software. The normal distribution was analyzed using Shapiro-Wilk or D'Agostino&Pearson tests (GraphPad Prism 8 software). The results were considered statistically significant when the *p*-value was less than 0.05 (^∗^), 0.01 (^∗∗^) or 0.001 (^∗∗∗^).

## Results

### Disruption of Hemidesmosomes in Prostate Epithelial Cells Promotes Their Migratory Activity

α6β4-integrin is a key component of HDs. We have recently established and characterized HD-defective benign (RWPE1) and malignant (PC3) prostate epithelial cells by depleting the expression of either α6- or β4-integrin subunit in these cell lines (Wenta et al., 2021). RWPE1 cell line derives from human papilloma virus 18-immortalized histologically normal prostate tissue (Bello et al., 1997) and PC3 cell line originates from bone metastasis of a grade IV prostatic adenocarcinoma (Kaighn et al., 1979). Both RWPE1 and PC3 cells express α6β4-integrins and form HD-like adhesions containing plectin and CD151 (Wenta et al., 2021). However, the pattern of HD-like adhesions was different, in benign RWPE1 cells α6β4-integrins covered nearly the entire basal surface, whereas in malignant PC3 cells, these adhesions were more restricted towards cell periphery (Wenta et al., 2021). Interestingly, it was observed that loss of α6- or β4-integrin expression in the respective cell lines enhanced their tumorigenic potential and cell migration capacity measured using scratch wound assay. To study the link between HDs and cell migration in more detail we first confirmed the depletion of respective integrin subunit expression in the cell lines to be analyzed (Supplementary Figure S1A). Second, we rechecked their cell migration capacity using the *in vitro* scratch wound assay. As reported earlier (Wenta et al., 2021), wounds in RWPE1 α6-KO and especially RWPE1 β4-KO monolayers recovered faster when compared with parental RWPE1 cells (Supplementary Figure S1B). The wound closure rates of PC3 α6-KO and PC3 β4-KO monolayers were also significantly faster than for parental PC3 monolayers (Supplementary Figure S1C) confirming the previous observation that loss of HDs promotes cell migration (Wenta et al., 2021).

**FIGURE 1:**
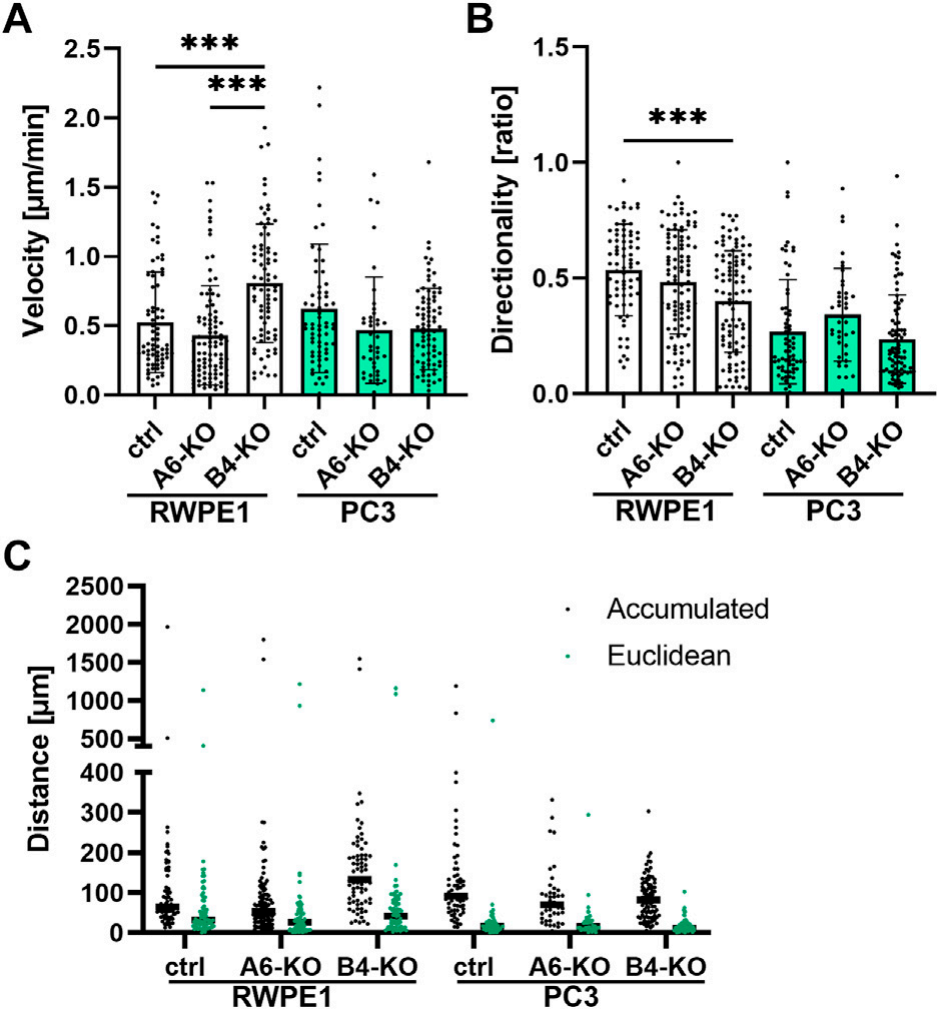
Loss of HDs modulates migratory properties of the cells. Single-cell tracking of sparsely seeded cells was applied to analyze the cell velocity **(A)**, directionality **(B)** and migration distance **(C)**. The graphs show the mean ± SD representing data combined from two independent experiments with at least 35 single cells analyzed in each. Statistical significance was determined using Kruskal-Wallis test followed by Dunn’s multiple comparisons test (GraphPad Prism 8 software) and *p*-values are indicated by asterisks; 0.05 (*), 0.01 (**), or 0.001 (***).

In epithelial cell cultures, the scratch wound assay measures mostly collective cell migration that is directed by cell-cell interactions but, at longer time frames, this assay is also influenced by cell proliferation. To focus on specific migration features such as velocity and directional persistence, HD-depleted cells were analyzed using single-cell tracking of sparsely seeded cells (Supplementary Videos S1–S6). In agreement with scratch wound assay results, the velocity of migrating RWPE1 β4-KO cells was markedly higher than RWPE1 or RWPE1 α6-KO cells (Figure 1A). The directionality of RWPE1 β4-KO cells was, however, impaired (Figure 1B). Whereas RWPE1 cells displayed robust front-rear polarity that was frequently maintained for several minutes and accompanied by persistent migration directed by the leading edge of the cell, RWPE1 β4-KO cells displayed frequent changes in the direction of migration (Figure 1B). Surprisingly, neither the velocity nor the directionality of RWPE1 α6-KO cells was significantly different from those of RWPE1 cells (Figures 1A,B).

As expected, the parental malignant PC3 cells displayed lower directionality than benign RWPE1 cells (Figure 1B). PC3 cells were also more actively migrating as evidenced by the longer accumulated distance traveled (Figure 1C). However, HD-depleted PC3 cells did not show significant differences in velocity compared with PC3 cells (Figure 1A). Curiously, the average migration velocity of PC3 α6-KO and PC3 β4-KO cells tended to be lower than in parental PC3 cells although the difference was not statistically significant (Figure 1A). For PC3 α6-KO cells, a non-significant tendency for increased directionality was observed but all three PC3 variants displayed similar Euclidean distances traveled (Figures 1B,C). Taken together, we found that loss of HDs modulates cell migration properties, but the overall effect on migration depends on the assay used and can be different depending on whether α6- or β4-integrin is depleted.

### Loss of Hemidesmosomes Stimulates Focal Adhesions Formation in Prostate Epithelial Cells

**FIGURE 2:**
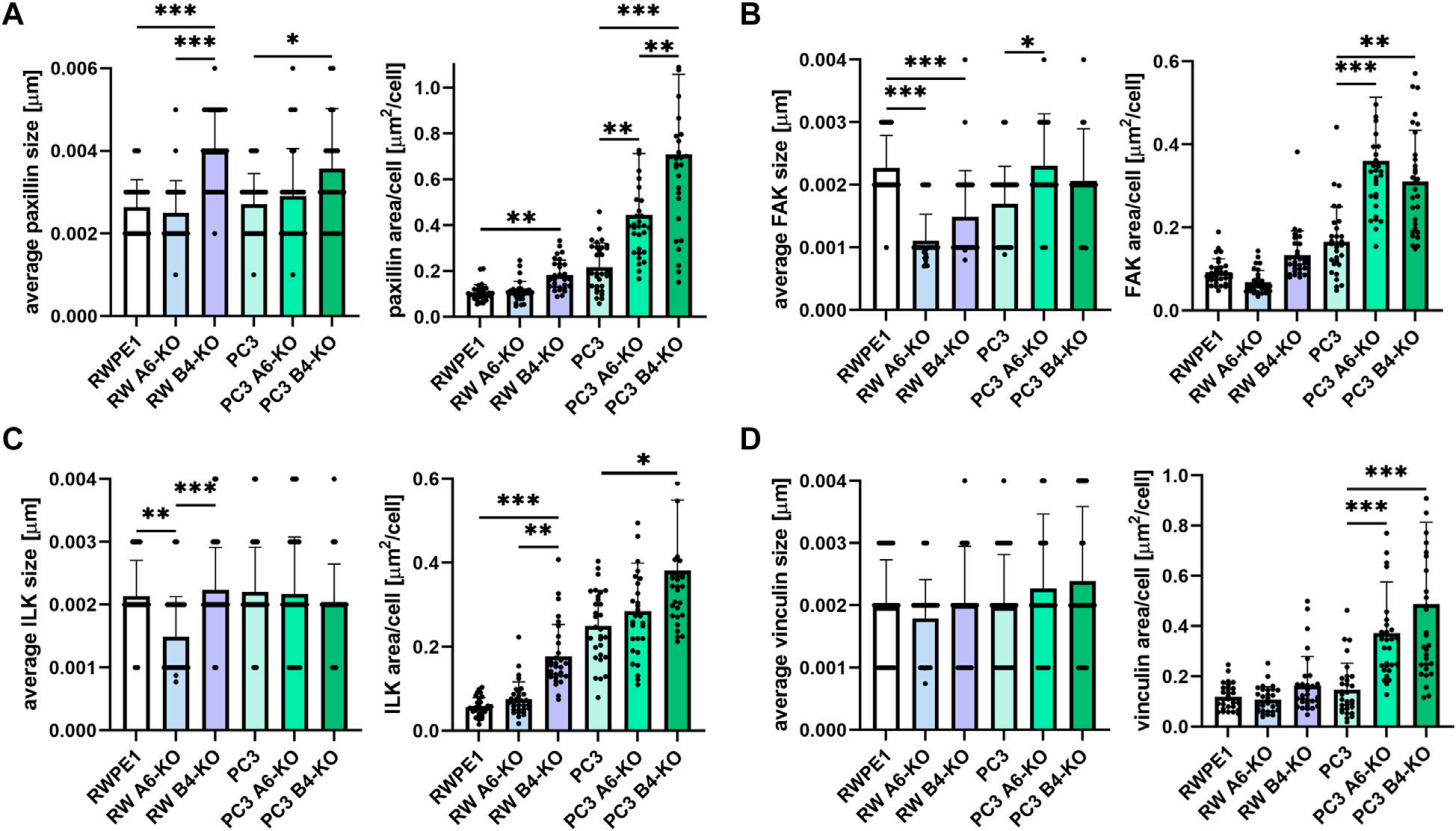
Disruption of HDs by depleting α6- or β4-integrin induces FAs formation. RWPE1, RWPE1 α6-KO, RWPE1 β4-KO, PC3, PC3 α6-KO and PC3 β4-KO cells were stained for FA markers: paxillin **(A)**, FAK **(B)**, ILK **(C)** or vinculin **(D)** and imaged using TIRF microscopy. The size of individual FAs and the total area covered by the indicated markers per cell were determined using Fiji/ImageJ software. A minimum of 150 RWPE1 or 70 PC3 cells were analyzed for each variant. Statistical significance was assessed with Kruskal-Wallis test followed by Dunn’s multiple comparisons test comparing separately RWPE1 to its HD-depleted derivatives and PC3 to PC3 α6-KOs and PC3 β4-KOs using GraphPad Prism 8 software. *p*-values lower than 0.05 (*), 0.01 (**), or 0.001 (***) were considered statistically significant.

Careful observation of the timelapse videos hinted that HD-depleted RWPE1 and PC3 cells might have increased lamellipodial activity even when no overall effect on cell migration velocity or directionality was seen (Supplementary Videos S1–S6). Cell migration is guided by temporally and spatially regulated cycles of FA formation, maturation, and disassembly (Khan and Goult, 2019; Shellard and Mayor, 2020). To assess the potential effects of HD-loss on FAs in prostate epithelial cells, we first analyzed the size and abundance of selected key FA proteins by immunofluorescence in the different cell variants (Figure 2; Supplementary Figure S2). Paxillin is a multidomain scaffolding protein that binds directly to the cytoplasmic tail of β1-integrins thereby contributing to integrin activation and FA formation (López-Colomé et al., 2017). The average size of paxillin foci was increased only in the β4-KO variants of both the RWPE1 and PC3 cells, but not in α6-depleted variants (Figure 2A). However, the number of paxillin foci was significantly increased also in the PC3 α6-KO cells. No significant differences in paxillin foci size or abundance were observed in RWPE1 α6-KO (Figure 2A). Focal adhesion kinase (FAK) is a tyrosine kinase that regulates integrin-mediated signaling and interacts with multiple integrin effectors, including paxillin (Murphy et al., 2020). In agreement with paxillin data, loss of HDs led to an increase in the number of FAK foci per cell, especially in PC3 α6-KO and PC3 β4-KO cells (Figure 2B). In contrast, the average size of FAK-foci was bigger only in PC3 α6-KO and PC3 β4-KO (not significant) cells whereas in HD-depleted RWPE1 cells FAK foci were significantly smaller than in control cells (Figure 2B). Integrin-linked kinase (ILK) is a pseudokinase adaptor protein that conveys growth-promoting signals from FAs (Tsirtsaki and Gkretsi, 2020). Except for RWPE1 α6-KO cells containing small ILK-foci, loss of HDs did not affect the size of ILK-positive foci (Figure 2C). Curiously, malignant PC3 cells contained significantly more ILK-positive foci when compared with benign RWPE1 cells. However, in both cell types, β4-KO caused a significant increase in ILK-positive foci whereas α6-KO led only to a modest increase (Figure 2C). Vinculin reinforces the linkage of FAs to actin cytoskeleton upon increased forces onto adhesions and vinculin recruitment is thus a key marker of force-induced FA maturation (Bays and DeMali, 2017). The size of vinculin foci was unchanged in all the cell types (Figure 2D). In HD-depleted RWPE1 variants the number of vinculin foci also remained unchanged (Figure 2D). In HD-depleted PC3 cells, however, a robust increase in the number of vinculin foci was observed (Figure 2D). In conclusion, we observed clear increase in the number of FAs in HD-depleted RWPE1 and PC3 cells. Although the changes seen in FA numbers and size were similar in α6- and β4-KOs, the effects were more robust in β4-integrin-depleted cells. The size of FA foci, particularly for the markers of mature adhesion was not changed. Overall, the effects of α6- or β4-KOs were more prominent in malignant PC3 cells that also had higher FA number when compared with benign RWPE1.

### Establishment and Characterization of Cell Models to Study Focal Adhesions Dynamics in Prostate Epithelial Cells

**FIGURE 3:**
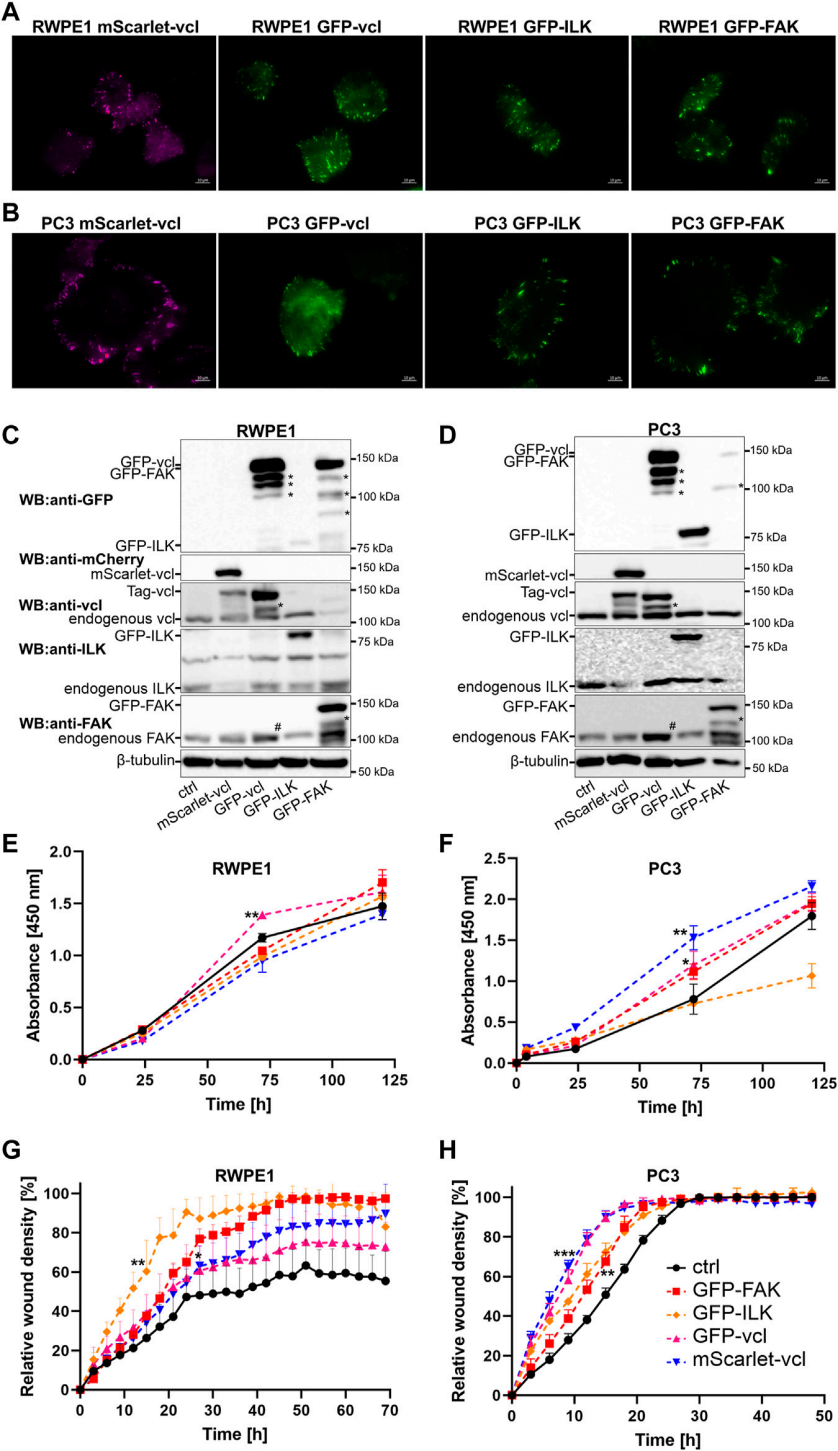
Establishment and characterization of RWPE1 and PC3 cells overexpressing fluorescence protein-tagged FA marker proteins. FACS-sorted populations of **(A)** RWPE1 and **(B)** PC3 cells stably transfected with mScarlet-vinculin (vcl), GFP-vcl, GFP-ILK or GFP-FAK constructs were imaged using TIRF microscopy. The expression levels of the indicated ectopically expressed fusion proteins in **(C)** RWPE1 and **(D)** PC3 cells were compared to endogenous expression levels by western blotting using antibodies recognizing the target protein. Fusion proteins were specifically detected using antibodies for GFP and mScarlet. The asterisks (*) denote the bands of proteolytically cleaved FA proteins. The symbol (#) indicates an increased FAK level in the cells expressing exogenous vinculin. **(E)** Parental RWPE1 and **(F)** PC3 cells and their respective derivatives overexpressing GFP-FAK, GFP-ILK, GFP-vinculin or mScarlet-vinculin were subjected to XTT-assay to measure cell proliferation. The data shows mean ± SD from at least two independent analyses performed in triplicates. The migratory properties of indicated variants of **(G)** RWPE1 and **(H)** PC3 cells were determined using the scratch wound assay module of IncucyteS3. The graphs show the mean ± SD. The analyses show a representative experiment out of three independent repeats with at least four replicates per variant. Statistical significance was determined using Two-way ANOVA followed by Dunnett’s multiple comparisons test (GraphPad Prism 8 software) and *p*-values are indicated by asterisks; 0.05 (*), 0.01 (**), or 0.001 (***).

To get a more detailed insight into the dynamics of FA formation and maturation we established and characterized a panel of cell populations stably expressing fluorescent protein-tagged exogenous FA proteins: GFP-FAK, GFP-ILK, mScarlet-vinculin and GFP-vinculin from ectopic promoters. All the markers displayed typical FA distribution (Figures 3A,B). Western blot analysis was done to monitor the relative expression levels of the fusion proteins compared with the respective endogenous proteins (Figures 3C,D). Most of the ectopically expressed constructs were overexpressed to a variable degree and we also observed additional proteolytically processed forms of vinculin- and FAK-fusions (Figures 3C,D, indicated by *). Of note, GFP-vinculin overexpression consistently led to overexpression of FAK in both RWPE1 and PC3 cells (Figures 3C,D indicated by ^#^). To assess the effect of FA-marker overexpression *per se* on epithelial cell proliferation and migration, an XTT (Figures 3E,F) and scratch wound (Figures 3G,H) assays were performed, respectively. We found that vinculin overexpression had a significant stimulatory effect on both proliferation and migration of RWPE1 and PC3 cells (Figures 3E–H). Overexpression of FAK and ILK promoted wound closure of RWPE1 and PC3 monolayers and stimulated the proliferation of PC3 cells. Substituting the GFP-tag in GFP-vinculin with monomeric red fluorescent protein mScarlet (Bindels et al., 2017) did not significantly affect the effects of vinculin overexpression on wound closure (Figures 3G,H; Supplementary Figure S3) although the expression levels of mScarlet-vinculin were lower than that of GFP-vinculin (Figures 3C,D). mScarlet-vinculin cells displayed a smaller effect on proliferation (Figures 3E,F) and showed no effect on FAK expression levels (Figures 3C,D).

**FIGURE 4:**
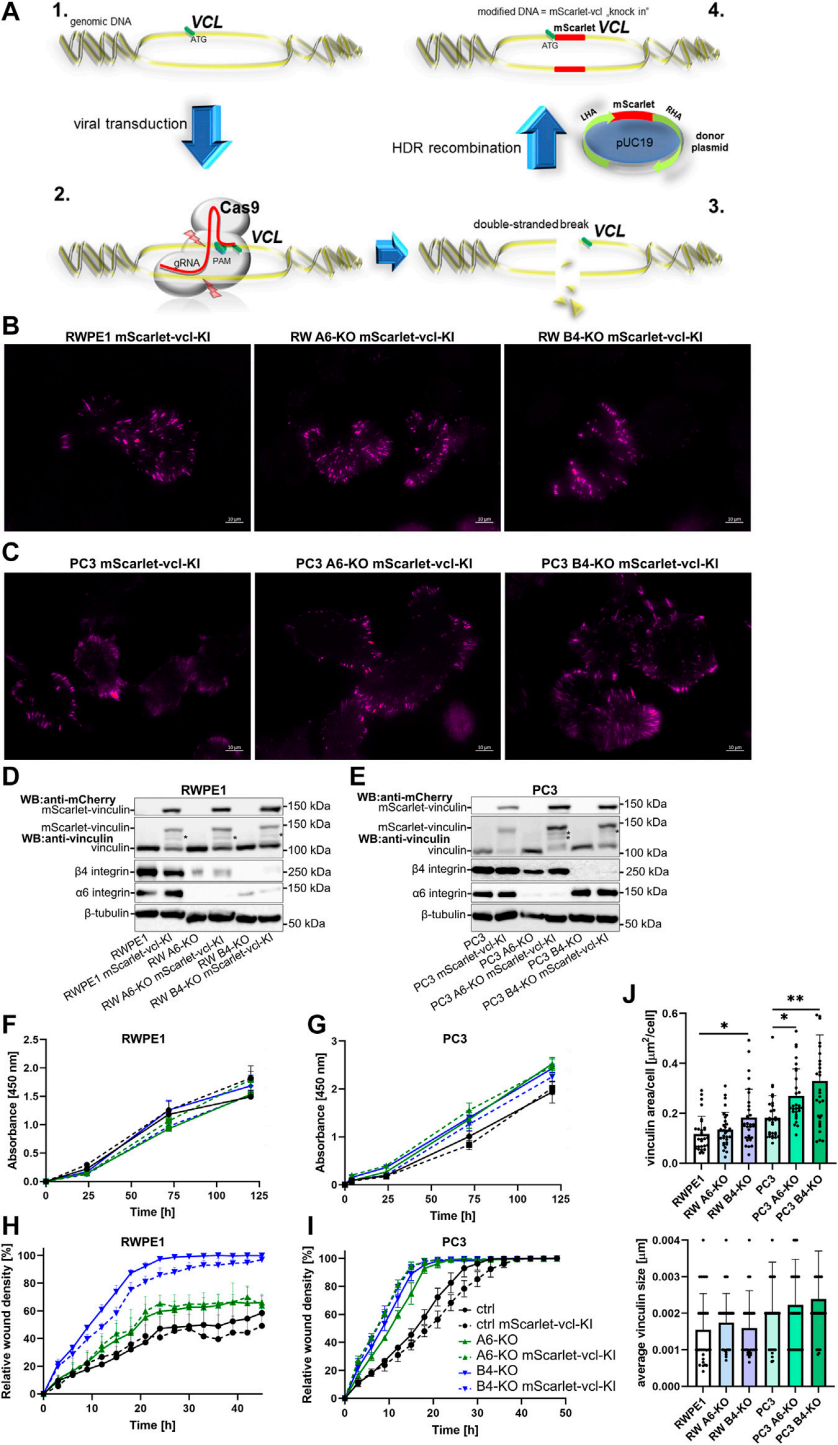
Generation and characterization of the RWPE1 and PC3 mScarlet-vinculin knock-in cell lines. **(A)** Schematic of the strategy of the in-frame *mScarlet* knock-in into *VCL* gene locus. 1—A gRNA construct targeting a sequence nearby the *ATG* start codon of *VCL* was designed and cloned into plentiCRISPRv2 vector. 2—Target cells were transfected with a donor vector containing *mScarlet* flanked by left (LHA) and right (RHA) homology arms followed by transduction with Cas9/gRNA-expressing lentiviral vector. 3—Targeted double-stranded DNA breaks at the *VCL* start site are repaired by homologous recombination with the donor construct resulting in endogenous in-frame fusion of *mScarlet* in front of the *VCL* gene (4). FACS-sorted populations of **(B)** RWPE1 and **(C)** PC3 mScarlet knock-in variants were imaged using TIRF microscopy. The expression levels of endogenous fusion proteins were determined by western blots in parental and HD-depleted variants of **(D)** RWPE1 and **(E)** PC3 cells. Fusion proteins were detected using mCherry antibodies. **(F)** Parental RWPE1 and **(G)** PC3 cells and their respective derivatives with endogenously tagged mScarlet-vinculin were subjected to XTT-assay to measure cell proliferation. The data shows mean ± SD from at least two independent analyses performed in triplicates. The migratory properties of indicated variants of **(H)** RWPE1-mScarlet-vinculin-KI and **(I)** PC3-mScarlet-vinculin-KI cells were determined using the scratch wound assay module of IncucyteS3. The graphs show the mean ± SD. The analyses show a representative experiment out of three independent repeats with at least five replicates per variant. Two-way ANOVA followed by Dunnett’s multiple comparisons test was applied for statistical analysis. **(J)** The size of individual FAs and the total area per cell covered by endogenous mScarlet-vinculin in the indicated cell lines were determined using Fiji/ImageJ software. A minimum of 150 RWPE1 or 70 PC3 cells were analyzed for each sample. Statistical significance was determined using Kruskal-Wallis test followed by Dunn’s multiple comparisons test (GraphPad Prism 8 software) and *p*-values are indicated by asterisks; 0.05 (*), 0.01 (**), or 0.001 (***).

These findings reveal a potential caveat in overexpression models where the high levels of overexpressed proteins might affect the process that is being studied. This seemed to be the case for vinculin. To address this issue, we made use of CRISPR-Cas9-mediated homology-directed repair approach to “knock-in” *mScarlet* into *VCL* gene in the genome of the different RWPE1 and PC3 cell variants. The resulting mScarlet-vinculin fusion protein is expressed under the control of an endogenous promoter. A schematic of the knock-in approach is presented in Figure 4A. All the cell variants were analyzed by TIRF microscopy (Figures 4B,C), western blotting (Figures 4D,E) and sequencing to confirm the correct incorporation of mScarlet coding sequence into the *VCL* gene. Western blot analysis revealed mostly mono-allelic incorporation of mScarlet. The expression levels were correlated with the endogenous levels from the WT-allele, but we still noted some proteolytic processing of the fusion protein (Figures 4D,E indicated by *). Importantly, cell proliferation and migration analysis of control and HD-depleted mScarlet-vinculin cells showed that endogenously tagged vinculin had minimal effects on the properties of parental cells (Figures 4F–I). Furthermore, analysis of the number and size of mScarlet-vinculin foci recapitulated the results obtained for endogenous vinculin foci stained by immunofluorescence (Figures 4J, 2D). The subtle differences in foci numbers and size could be due to *mScarlet* knock-in affecting only one of the two alleles and the observed processing of the fusion protein (Figures 4D,E).

### Loss of Hemidesmosomes Stimulates Focal Adhesions Dynamics in Prostate Epithelial Cells

**FIGURE 5:**
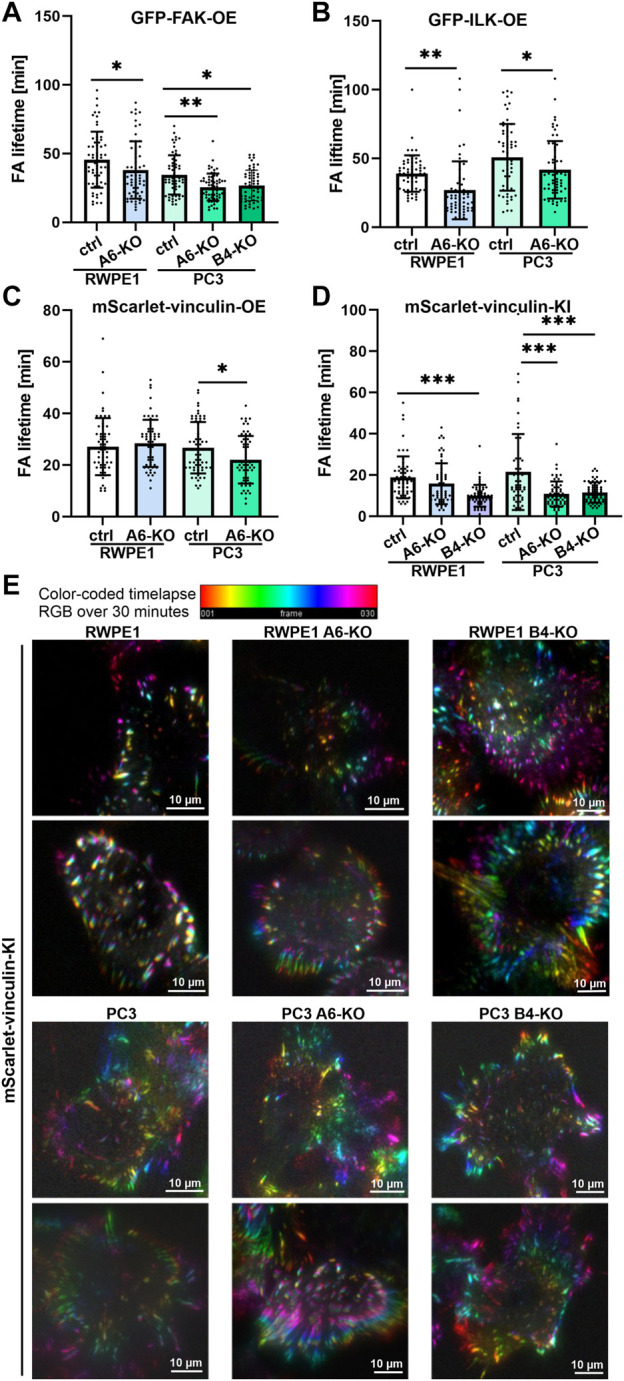
HDs depletion induces FA dynamics. Control and HD-deficient (α6- or β4-integrin-depleted) RWPE1 and PC3 cells overexpressing fluorescence FA proteins were seeded onto fibronectin-coated glass-bottom culture dishes and imaged at 1 min intervals using TIRF microscopy. The lifetime of individual **(A)** GFP-FAK, **(B)** GFP-ILK and **(C)** GFP-vinculin positive FAs was determined from recorded time-lapse series. **(D)** Lifetimes of foci formed by endogenously expressed mScarlet-vinculin were determined in RWPE1, RWPE1 α6-KO, RWPE1 β4-KO, PC3, PC3 α6-KO and PC3 β4-KO cells lines as described above. A minimum of 60 FAs were analyzed for each variant in A-D. Statistical significance was assessed with Kruskal-Wallis test followed by Dunn’s multiple comparisons test using GraphPad Prism 8 software [0.05 (*), 0.01 (**), or 0.001 (***)]. **(E)** A representative color-code timelapse of endogenously expressed mScarlet-vinculin foci in the indicated cell lines.

Next, we utilized the characterized cell models to study FA formation and disassembly in live cells. Control and HD-deficient (α6- or β4-integrin-depleted) RWPE1 and PC3 cells stably expressing GFP-FAK, ILK-GFP and mScarlet-vinculin from ectopic promoters were seeded onto fibronectin-coated coverslips and allowed to settle for 12 h before they were imaged at one-minute intervals using TIRF microscopy. The lifetime of individual foci was tracked and determined from the assemble time-lapse movies. Depletion of α6- or β4-integrins led to small but significant decreases in the lifetime of GFP-FAK (Figure 5A) in PC3 cells. Similar effect was observed in the lifetime of ILK-GFP (Figure 5B) and mScarlet-vinculin (Figure 5C) in both RWPE1 (apart from overexpressed mScarlet-vinculin) and PC3 cells indicating faster FA turnover upon HD disruption. Since overexpression of all the constructs, particularly vinculin, was found to influence wound closure kinetics, we validated this observation by using endogenously tagged vinculin cell lines that did not show altered cell migration (Figures 4H,I). mScarlet-vinculin knock-in was established in RWPE1, RWPE1 α6-KO, RWPE1 β4-KO, PC3, PC3 α6-KO and PC3 β4-KO cells. The different mScarlet-vinculin knock-in cell variants were seeded and analyzed as described above. In line with the data from vinculin overexpressing cells, knock-in mScarlet-vinculin showed shorter FA lifetimes (Figure 5D). The effect was bigger than in the overexpression model, although the difference was again statistically significant only in PC3 cells (Figures 5C,D). However, β4-integrin depletion robustly induced vinculin turnover and dynamics in both PC3 and RWPE1 cells (Figure 5D). Faster dynamics of FAs in HD-depleted cells were visualized by generating color-coded timelapse overlays (Figure 5E). Taken together, our data show that HDs depletion affects the FA turnover dynamics by inducing the assembly of FAs that, however, have shorter lifetimes when compared with FAs in a cell with intact HDs.

**FIGURE 6:**
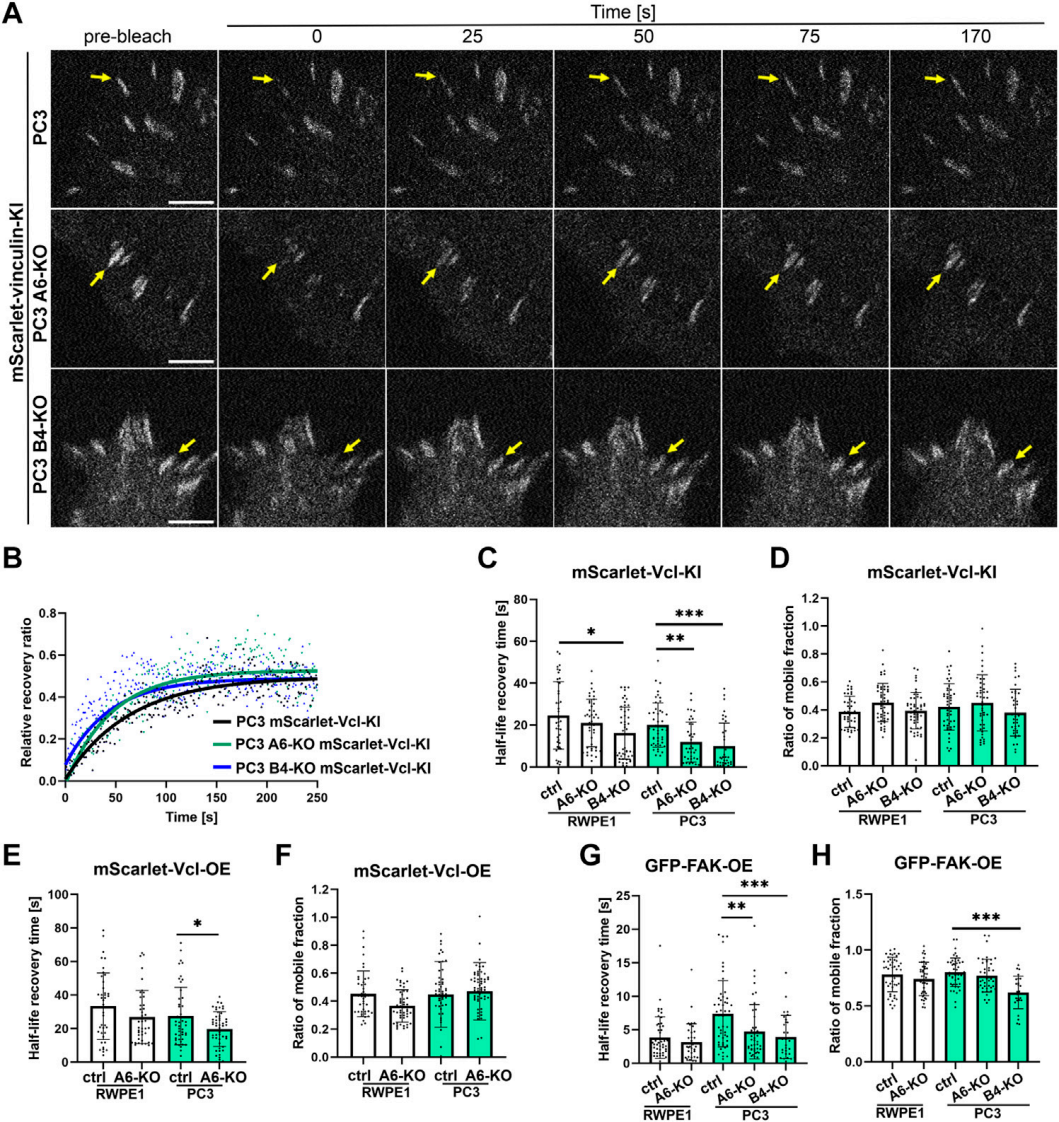
Loss of HDs affects the diffusion coefficients of FA components. Representative pictures of bleached regions of endogenous mScarlet-vinculin foci **(A)** and plots of relative fluorescence recovery ratio of that protein in the function of time in PC3 cell variants **(B)** the analysis of half-life recovery times and ratio of mobile fractions of bleached endogenous mScarlet-vinculin foci [**(C)** and **(D)**, respectively], exogenous mScarlet-vinculin [**(E)** and **(F)**, respectively] and exogenous GFP-FAK [**(G)** and **(H)**, respectively] in RWPE1 and PC3 cells. The data are represented as a mean ± SD from at least 50 individual FAs from at least 10 random cells. Statistical significance was analyzed by Kruskal-Wallis test followed by Dunn’s multiple comparisons test using GraphPad Prism 8 software [0.05 (*), 0.01 (**), or 0.001 (***)].

To analyze FA dynamics in more detail we used confocal microscopy to perform fluorescence recovery after photobleaching (FRAP) experiments to study intracellular dynamics of FAK and vinculin. FAK is an important signaling protein that has been shown to rapidly diffuse in and out of FAs whereas vinculin is a structural mechanoresponsive FA-protein whose diffusion rates are slower and further stabilized upon increased actin-crosslinking as FAs mature (Stutchbury et al., 2017). We first analyzed molecular dynamics of endogenously tagged vinculin in tumorigenic PC3 cells which upon HD disruption displayed significantly shortened FA lifetimes. mScarlet-Vinculin recovery rates after photobleaching were significantly faster in both PC3 α6-KO and PC3 β4-KO cells when compared with PC3 controls indicating faster diffusion (Figures 6A–D). No significant changes were observed in the ratio of mobile fractions of mScarlet-vinculin in the different PC3 cell variants (Figure 6D). Similar analysis of mScarlet-vinculin in benign RWPE1 cells revealed significantly faster diffusion kinetics only in RWPE1 β4-KO cells again suggesting that loss of β4-integrin leads to more robust phenotypes (Figure 6C). To compare the molecular dynamics of overexpressed protein with the endogenously expressed version we next performed FRAP analysis in wild-type and α6-integrin depleted PC3 and RWPE1 cells overexpressing mScarlet-vinculin. In agreement with the vinculin KI-data, overexpressed vinculin showed faster diffusion rates in α6-depleted PC3 cells whereas the effect was not statistically significant in RWPE1 variants (Figure 6E). No effects were seen in the mobile fraction of mScarlet-vinculin in any of the cell lines (Figure 6F). Finally, we looked at the molecular dynamics of overexpressed FAK in these cells and found that, as reported earlier, FAK was much more mobile and had faster recovery rates than vinculin (Figures 6G,H). Nevertheless, FAK diffusion was still significantly faster in PC3 α6-KO and β4-KO cells when compared with parental PC3 cells (Figure 6G). No significant effect has been seen in the mobile fraction of FAK in PC3 α6-KO but reduction of the ratio of mobile fraction was found in PC3 β4-KO (Figure 6H). In RWPE1 cells we did not observe statistically significant changes in GFP-FAK dynamics (Figures 6G,H). It is possible that activation of FA dynamics upon loss of HDs is more prominent in tumorigenic PC3 cells than in RWPE1 due to the absence of PTEN which was reported to synergistically promote FA-mediated signaling in HD-depleted prostate cancer cells (Wenta et al., 2021).

## Discussion

HDs and FAs co-exist in epithelial cells to mediate cell-ECM adhesion and HDs are thought to mediate stable cell anchorage to the basement membrane while FAs have been reported to be dynamic adhesions that regulate cell migration. In agreement with the role of HDs in mediating robust adhesion and the integrity of epithelium, *ITGB4*- and *ITGA6*-knock-out mice die soon after birth due to extensive detachment of squamous epithelia from the basement membranes (Dowling et al., 1996; Georges-Labouesse et al., 1996; Neut et al., 1996). However, it is clear that both HDs and FAs are dynamic structures (Elaimy et al., 2019; Pora et al., 2019). Proteomics analysis of HDs and FAs have revealed that while they are distinct structures, they share a few scaffold complexes potentially linking the two adhesions (Horton et al., 2015; Myllymäki et al., 2019; te Molder et al., 2020). Indeed, the composition of FA and HD dynamics appear to be co-regulated in migrating epithelial cells involving a yet incompletely defined signaling crosstalk (Hopkinson et al., 2014; van Bodegraven and Etienne-Manneville, 2020; Moch and Leube, 2021; te Molder et al., 2021). While the role of FA dynamics in cell migration has been extensively studied the role of HDs is still less well understood (van Bodegraven and Etienne-Manneville, 2020).

Recently, in a study addressing the role of HDs in prostate cancer we made an observation that α6- or β4-integrin depleted prostate epithelial cells had faster wound closure kinetics (Wenta et al., 2021). Here, by using both different ectopically overexpressed FA-markers and endogenously tagged vinculin, we report that disruption HDs promotes cell migration by influencing FA dynamics. These findings are in line with the reported role of type I HDs in keratinocytes (Ozawa et al., 2010). All the α6- and β4-integrin-depleted normal and tumorigenic cells displayed faster wound closure kinetics. Interestingly, although depletion of either α6- or β4-integrin expression leads to disassembly of HDs in prostate epithelial cells, we observed also important differences between α6- and β4-KO phenotypes. In most of the analyses, cells lacking β4-integrin had stronger phenotypes compared with α6-KOs. Moreover, when single-cell migration was studied, only RWPE1 β4-KO cells displayed significantly faster migration velocity although both RWPE1 β4- and α6-KOs showed a tendency for defective establishment of front-rear polarity. Curiously, keratin-5 filament organization is lost in RWPE1 β4-KO cells while in RWPE1 α6-KO cells it is partially retained despite significantly reduced β4-integrin expression at the basal surface in these cells (Supplementary Figure S2 and unpublished observation). In contrast, significant α6-integrin staining remained at the basal surface of β4-KO cells in which α6-integrin accumulates in the proximity of FAs, especially in the malignant PC3 background (Supplementary Figure S2). Interestingly, in malignant PC3 cells β4-integrin is still expressed (Supplementary Figure S1A), although it is removed from the basal surface and the remaining staining appears to be mostly intracellular (Supplementary Figure S2). This could contribute to the observed improved directionality and decreased velocity of PC3 α6-KO when compared with parental PC3 controls (Figures 1A,B). It remains to be determined if this pool of β4-integrin contributes to migration or tumorigenesis but it is noteworthy, that the cytoplasmic tail of β4-integrin has been shown to interact with plectin in the absence of α6β4-integrin heterodimer formation (Nievers et al., 1998).

In addition to inducing FAs formation, disruption of HDs also facilitated FAs turnover rates. Such phenotype could have different effects on cell migration depending on assay conditions. The scratch wound assay measures mostly collective cell migration in epithelial cultures although malignant cancer cells can also migrate individually into wounds. In any case, cell-cell contacts can have a major influence on cell migration as was frequently observed for cells encountering each other in sparse cultures used for single-cell tracking experiments (Supplementary Videos S1–S6). Cells at the wound edge have only limited directional freedom to migrate into the wound whereas in single-cell tracking setup cells can freely migrate to all directions. It is thus possible that in the scratch wound assay, limited directional freedom combined with enhanced FA dynamics and lamellipodial activity could promote wound closure whereas in single-cell tracking non-polarized lamellipodial activity would not facilitate migration due to the formation of lamellipodia to opposite directions. Further studies are needed to address these possibilities.

It has been reported that, under some circumstances, α6β4-integrins may regulate cell migration by interacting with the actin cytoskeleton (Rabinovitz and Mercurio, 1997). However, this function is not thoroughly understood and might also be indirect depending on the ability of α6β4-integrins to activate various growth factor signaling pathways possibly regulating FA-associated integrins (Shaw, 2001; Lipscomb et al., 2003). Our data support the idea that at least in subconfluent cells, α6-integrin is targeted to actin cytoskeleton in the absence of β4-integrin, likely forming α6β1-heterodimers (Colburn and Jones, 2017). A study in kidney epithelial cells also showed that α6-integrin reaches the cell surface in the absence of either β4- or β1-integrin but not when both of them are deleted. A recent study also demonstrated that loss of an intact α6β4-integrin/plectin linkage led to increased FA formation, cell spreading and traction-force generation (Wang et al., 2020). Interestingly, HD-assembly was shown to regulate FA-targeting of αVβ5-integrins revealing another possible mechanism of how HDs regulate FA-dynamics. It is worth to note that α6β4-integrin has been implicated in the regulation of the expression levels of FA-associated integrins (Kligys et al., 2012).

Prostate cancer progression is accompanied by loss of HDs organization and particularly the loss of β4-integrin expression (Knox et al., 1994; Allen et al., 1998; Davis et al., 2001). However, the effect of HD depletion might be context-dependent as β4-integrin has also been reported to induce expansion of prostate tumor progenitors (Yoshioka et al., 2013). HD organization could also be disrupted without loss of β4-integrin for example via alternative splicing or posttranslational modifications of α6β4-integrins (Demetriou et al., 2004; Pawar et al., 2007; Wang et al., 2019). Interestingly, loss of β4-integrin has been shown to lead to aberrant prostate glandular morphogenesis resembling invasive collective migration and increased FAK phosphorylation in prostate epithelial cells (Wang et al., 2017; Wenta et al., 2021). Here we found that FAK molecular dynamics were upregulated especially in β4-deficient cells leading to stimulated cell migration. How β4-integrin at HDs might inhibit FAK in FAs, is an interesting topic for future studies. In conclusion, we show that disruption of HDs and especially loss of β4-integrins, stimulates FA dynamics thereby facilitating the migration of prostate epithelial cells.

## Data Availability

The original contributions presented in the study are included in the article/Supplementary Material, further inquiries can be directed to the corresponding authors.

## Abbreviations

HD—hemidesmosome; FA—focal adhesion; FRAP—fluorescence recovery after photobleaching; TIRF—total internal reflection fluorescence; ECM—extracellular matrix; FAK—focal adhesion kinase; ILK—integrin-linked kinase; A6/B4-KO—α6-/β4 integrin knock-out; vcl-KI—vinculin knock-in; OE—overexpression; ctrl—control (wild type) cells.
