# Supplementary material for: Loss of α6β4 Integrin-Mediated Hemidesmosomes Promotes Prostate Epithelial Cell Migration by Stimulating Focal Adhesion Dynamics
Source: Front Cell Dev Biol. 2022 Jul 7;10:886569. doi: 10.3389/fcell.2022.886569 (PMC9301336; doi:10.3389/fcell.2022.886569)
Supplement: Supplementary file 6 [file DataSheet1.docx]

# Supplementary data

Loss of α6β4 integrin-mediated hemidesmosomes promotes prostate epithelial cell migration by stimulating focal adhesion dynamics

Anette Schmidt^1^, Mika Kaakinen^2^, Tomasz Wenta^1,3†^, Aki Manninen^1†^

^1^ Disease Networks Research Unit, Faculty of Biochemistry and Molecular Medicine, Biocenter Oulu, University of Oulu, Oulu, Finland

^2^ Oulu Center for Cell-Matrix Research, Faculty of Biochemistry and Molecular Medicine, Biocenter Oulu, University of Oulu, Oulu, Finland

^3^ Department of General and Medical Biochemistry, Faculty of Biology, University of Gdansk, Gdansk, Poland

† These authors have contributed equally to this work and share the last authorship:
Aki Manninen: [aki.manninen@oulu.fi](mailto:aki.manninen@oulu.fi)

Tomasz Wenta: [tomasz.wenta@ug.edu.pl](mailto:tomasz.wenta@ug.edu.pl)


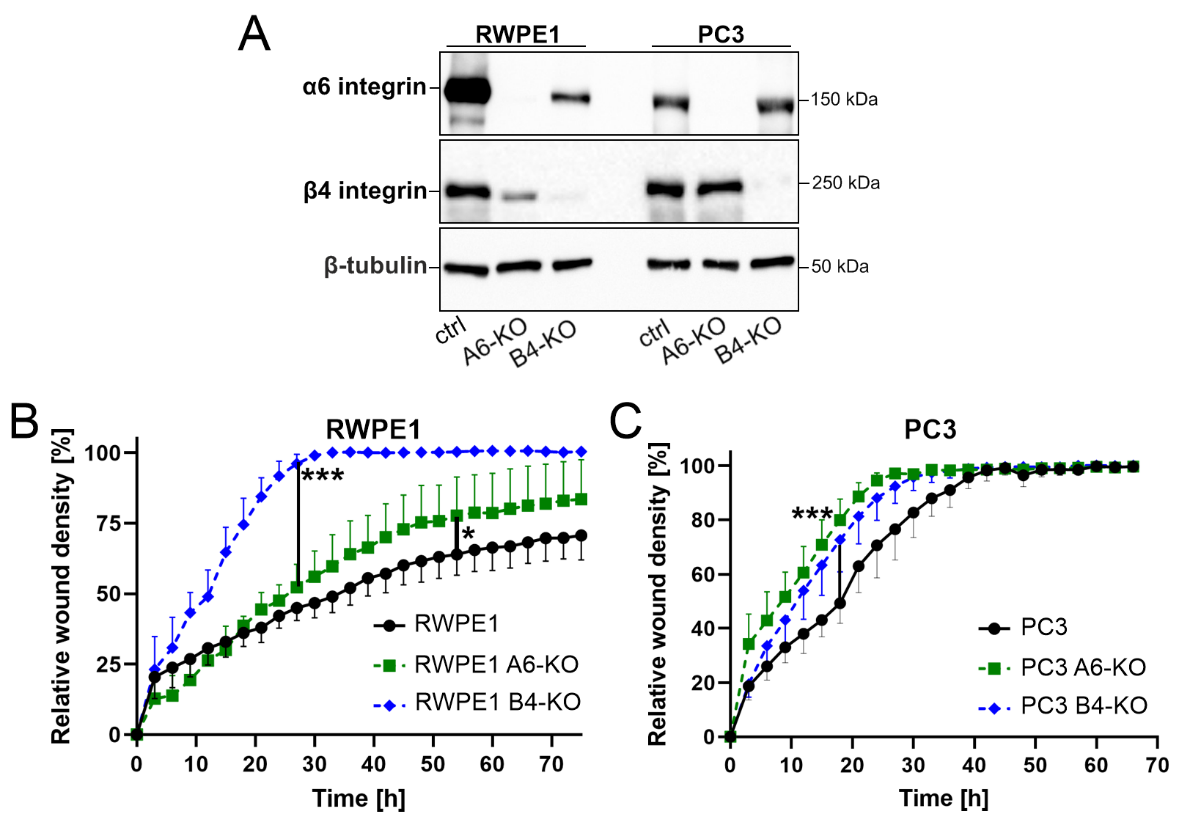


**Fig S1. Loss of hemidesmosomes stimulates cell migration.** (A) Western blotting analysis of RWPE1 and PC3 cells with depletion of α6 or β4-integrin. The migratory capacity of control and α6 or β4-integrin-depleted RWPE1 (B) and PC3 (C) cell variants was determined using the scratch wound assay module of IncucyteS3 as described in Wenta et al. 2021. The analyses show a representative experiment out of three independent repeats with at least five replicates per variant. The data is presented as a mean ± SD. Two-way ANOVA followed by Dunnett’s multiple comparisons test was applied to analyze the statistical significance in selected time points (marked on the plots). p-value * < 0.05, ** < 0.01, *** < 0.001 was considered statistically significant.


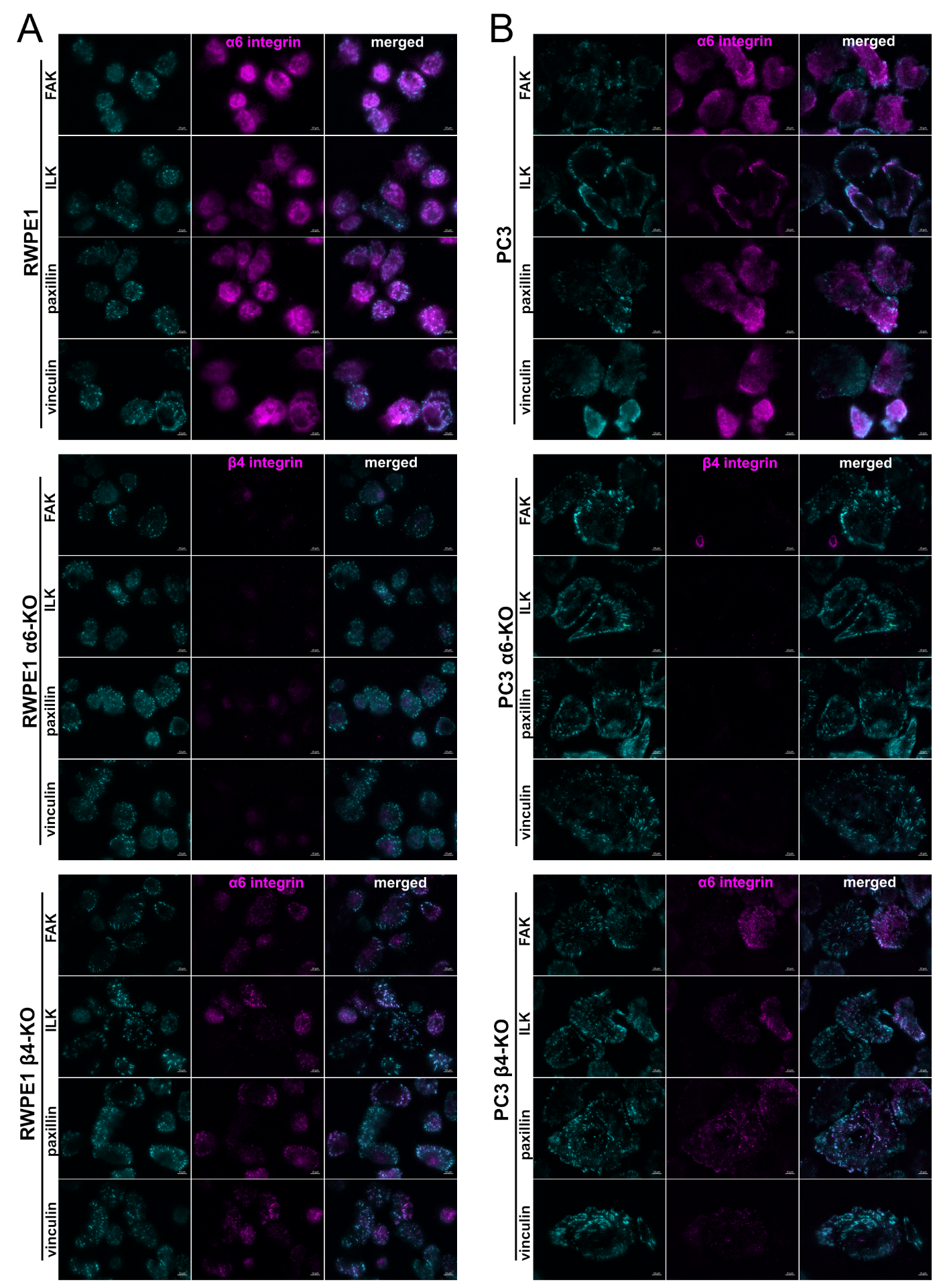


## Fig S2. Disruption of HDs in RWPE1 and PC3 cells by depleting α6- or β4-integrin expression induces formation of FAs. (A) The indicated RWPE1 and (B) PC3 cell lines were grown on glass-bottom culture dishes, fixed and stained for endogenous FAs markers paxillin, FAK, ILK or vinculin. Cells were imaged using TIRF microscopy. Quantitative analysis of size and number of the foci stained by the different FA markers is presented in Fig 2.


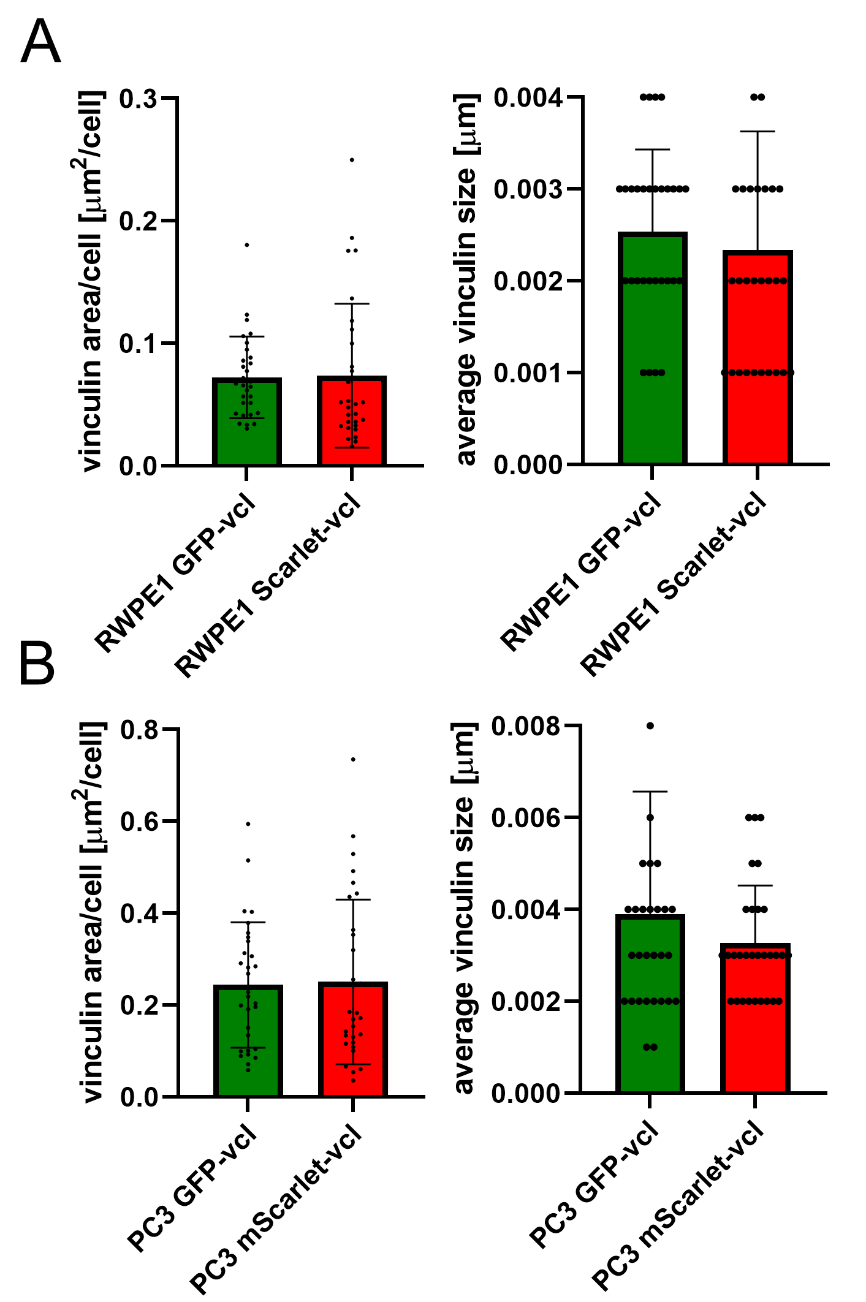


**Fig S3. GFP-vinculin and mScarlet-vinculin fusions with different levels of overexpression relative to endogenous vinculin show no differences in the size or number of vinculin-positive FAs.** (A) RWPE1 and (B) PC3 cells overexpressing GFP-vinculin or mScarlet-vinculin were seeded onto glass-bottom culture dishes and allowed to settle for 24 hours followed by imaging with TIRF microscopy. The size of individual FAs and the total area per cell covered by mScarlet- or GFP-vinculin was determined using Fiji/ImageJ software. A minimum of 150 of RWPE1 or 70 of PC3 cells were analyzed for each sample. Statistical significance was determined using One-way ANOVA followed by Sidak’s multiple comparisons test (GraphPad Prism 8 software). p-values were above 0.05 indicating no statistically significant differences.

## Table S1. Details of the plasmids used in this study.

| Name | Backbone | Insert | Source |
| --- | --- | --- | --- |
| pBabe_mScarlet-vcl | pBabe_puro | the sequence of *VCL* from *Gallus gallus* fused with *mScarlet* at the N-terminus inserted via *EcoR*I | This work |
| GFP-vinculin-PMXS | pMXS_puro | the sequence of *VCL* from *Gallus gallus* fused with GFP at the N-terminus | (Pietila et al., 2012) |
| ILK-GFP | pEGFP-C2 | the sequence of *ILK* fused with EGFP at the N-terminus | (Zhang et al., 2002) |
| pMXs-puro-EGFP-FAK | pMXS_puro | the sequence of *PTK2* from *Mus musculus* fused with *EGFP* at the N-terminus | Addgene #38194 (Hara et al., 2008) |
| pUC19_mScarlet_vcl_KI | pUC19 | the sequence of 877 bp upstream of *VCL* start codon fused with *mScarlet* and sequence of 927 bp of downstream *VCL* start codon inserted via *BamH*I | This work |

## Table S2. List of antibodies used in this study.

| **Name** | **Company** | **Cat. No** | **Dilution for WB** | **Dilution for ICC** |
| --- | --- | --- | --- | --- |
| anti-FAK | BD Transduction Laboratories | 610088 | 1:1000 | 1:50 |
| anti-GFP | Santa Cruz Biotechnology | sc-9996 | 1:500 |  |
| anti-ILK | Invitrogen | MA5-17228 | 1:1000 | 1:1000 |
| anti-mCherry | Abcam | ab183626 | 1:1000 |  |
| anti-paxillin | BD Transduction Laboratories | 610051 | 1:1000 | 1:100 |
| anti-vinculin | NovusBio | NB600-1293 |  | 1:100 |
| anti-vinculin | Sigma | V9131 | 1:1000 |  |
| anti-α6 integrin | Sigma | HPA012696 | 1:2000 |  |
| anti-α6 integrin | BD Transduction Laboratories | 555734 |  | 1:200 |
| anti-β4 integrin | Abcam | ab29042 |  | 1:100 |
| anti-β4 integrin | Santa Cruz Biotechnology | sc-9090 | 1:1000 |  |
| anti-β4 integrin | Abnova | MAB1371 |  | 1:250 |
| anti-β-tubulin | Sigma | T4026 | 1:5000 |  |
